# Airway microbial compartmentalization under mechanical ventilation: a randomized pilot study comparing an endotracheal tube with suction and continuous cuff pressure control (Venner PneuX^®^) to standard intubation

**DOI:** 10.1186/s12931-026-03849-2

**Published:** 2026-08-01

**Authors:** Anna E. Pilkowski, Patrick Schaal, Lienhard Leibold, Benjamin Seybold, Judith Schenz, Alexander H. Dalpke, Markus A. Weigand, Bachar A. Cheaib, Sébastien Boutin, Mascha O. Fiedler-Kalenka

**Affiliations:** 1 Medical Faculty Heidelberg, Department of Anesthesiology, Heidelberg University, Heidelberg, Germany; 2 Medical Faculty Heidelberg, Department of Infectious Diseases, Medical Microbiology and Hygiene, Heidelberg University, Heidelberg, Germany; 3 Institute of Medical Microbiology, University of Lübeck, Lübeck, Germany; 4 Translational Lung Research Center Heidelberg (TLRC-H), Member of the German Center for Lung Research (DZL), Heidelberg, Germany; 5 German Center for Infection Research (DZIF), Partner Site Heidelberg, Heidelberg, Germany; 6 Airway Research Center North (ARCN), German Center for Lung Research (DZL), Lübeck, Germany; 7 German Center for Infection Ressearch (DZIF), Partner Site Hamburg-Lübeck-Borstel-Riems, Lübeck, Germany

**Keywords:** Ventilator-associated pneumonia, Pulmonary microbiome, Endotracheal tube, Subglottic suction, Venner PneuX^®^

## Abstract

**Objectives:**

Ventilator-associated pneumonia (VAP) is driven in part by microaspiration along the endotracheal tube cuff, a process difficult to measure directly in ventilated patients. Because microbial communities differ between airway compartments, changes in their similarity over time may serve as an indirect readout of microaspiration. We assessed whether the Venner PneuX^®^ Tube (VT) system, combining cuff-pressure monitoring, subglottic suction, and a biofilm-resistant coating, reduces microbial exchange between airway compartments compared with a standard endotracheal tube (ST).

**Methods:**

In a prospective, randomized, single-center pilot study, 50 adults with acute respiratory failure received an ST or VT intubation. Microbial communities from five airway niches (throat, tracheal secretions, right upper, right lower, and left lower lung lobes) were sampled by 16 S rRNA sequencing at intubation (T1), after four days of ventilation (T2) and, for the tube tip, at extubation (T3). The primary outcome was the change in beta diversity (Morisita–Horn distances) between tube-associated, upper-airway, and lower-airway communities from T1 to T2.

**Results:**

Twenty-one of 50 randomized patients had complete microbiota data sets (9 ST, 12 VT) and were comparable in demographics, comorbidities, severity, and ventilation duration. In the ST group, tube-associated communities became more similar to tracheal and lower-airway communities from T1 to T2 (e.g. TS–Tube Morisita–Horn 0.55 → 0.30, *p* = 0.001), while lung regions diverged from each other and from the throat (LLL–LRL 0.08 → 0.31, *p* = 0.003; LLL–Throat 0.30 → 0.61, *p* < 0.001). None of these distances changed significantly in the VT group. Lower-airway Shannon diversity declined in both groups, more in the VT group.

**Conclusions:**

Standard intubation produced progressive microbial convergence between airway compartments, while the VT system did not. The findings provide biological plausibility for previously reported VAP reductions with the VT system and show that microbiota sampling can detect device-related differences in airway community structure, warranting further investigation as an endpoint for evaluating airway devices.

**Trial registration:**

The study is registered in the German Clinical Trials Register (DRKS- Deutsches Register für klinische Studien) under the clinical trial number: DRKS00029176. The Date of Trial Registration was 07.07.2022.

**Supplementary Information:**

The online version contains supplementary material available at https://doi.org/10.1186/s12931-026-03849-2.

## Introduction

Ventilator-associated pneumonia (VAP), defined by onset after ≥ 72 h of mechanical ventilation, is most prevalent in intensive care units and is associated with increased morbidity, mortality, and length of stay [1–4].

Culture-independent sequencing has refuted the assumption of a sterile lung and shown that distal airway communities are maintained largely by immigration from the upper airways, balanced by mucociliary clearance [5–7]. During mechanical ventilation, this balance is disrupted: pathogens can bypass the endotracheal tube cuff through microaspiration [8, 9], shifting the lower airway community toward the upper airway [6, 10] and tube-associated microbiota [10, 11]. Microaspiration is difficult to quantify directly in mechanically ventilated patients: in vitro cuff-leakage tests capture only fluid passage under controlled conditions [12], while clinical VAP surveillance detects only the end-stage consequence of cumulative aspiration [8, 13]. Microbial communities, in contrast, shift in composition in response to this flux, offering an indirect but continuous biological signature of exchange between airway compartments [7, 14, 15].

The Venner PneuX^®^ Tube (VT) system is designed to prevent VAP through a fold-free silicone cuff with continuous pressure monitoring, subglottic suction, and a biofilm-resistant inner coating [16]. Because the lung microbiota offers a sensitive readout of microaspiration [14], it provides a physiologically relevant way to assess whether such device modifications alter microbial exchange between airway compartments – a question that has not been studied. If microbial compartmentalization is indeed better preserved with the VT system, this would provide biological plausibility for the reduced VAP incidence reported in previous clinical studies [16, 17] and support the use of microbiota-based readouts for evaluating airway device performance.

## Materials and methods

### Study design and participants

This prospective, randomized, single-center pilot study was conducted in the Surgical Intensive Care Unit of Heidelberg University Hospital between November 2022 and May 2024 (DRKS00029176). Fifty adults with acute respiratory failure requiring endotracheal intubation were enrolled and randomized to receive either an ST tube (*n* = 25) or the VT system (*n* = 25) using sealed envelopes stored on the airway management trolley. Written informed consent was obtained from patients or legal representatives. Full inclusion and exclusion criteria are listed in the Supplementary Methods.

### Sample collection

**Fig. 1:**
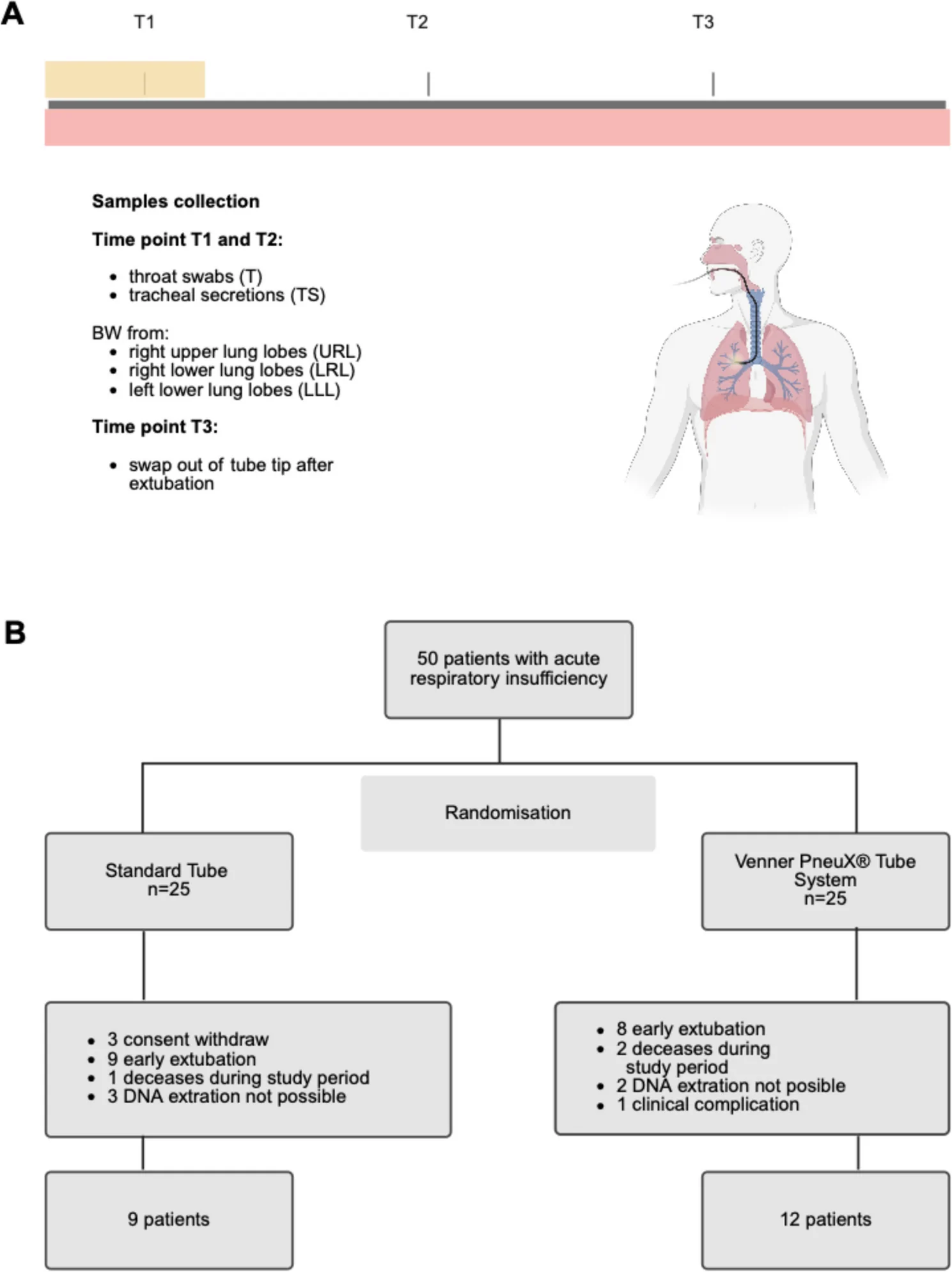
**A**: Samples Collection at three time points: T1: Day of intubation, T2: 72–96 h after intubation, T3: Day of extubation (passed spontaneous awakening trial (SAT) and passed spontaneous breathing trial (SBT)). BW = bronchial wash. **B**: Flowchart illustrating the patients included in the study and the reasons for incomplete datasets [18]

Samples were collected at three timepoints (Fig. 1A). At intubation (T1) and after four days of mechanical ventilation (T2), throat swabs (T), tracheal secretions (TS), and bronchoalveolar wash (BW) from the right upper (URL), right lower (LRL), and left lower (LLL) lung lobes were obtained in standardized order to minimize cross-contamination, using 10 mL 0.9% NaCl per BW site. Between each sampling step, the working channel of the bronchoscope was flushed with saline to minimize the risk of cross-contamination. At extubation (T3), the tube tip was sampled to characterize the tube-associated microbial community. Clinical severity scores were recorded at all three timepoints.

### Patient inclusion in analysis

Of the 50 randomized patients, 3 were excluded before analysis (withdrawal of consent), leaving 47 for demographic and clinical analyses. Of these, 21 patients (9 ST, 12 VT) had complete microbiota datasets across all niches at both T1 and T2 and were included in the primary microbiota analysis (Fig. 1B).

### DNA sequencing

DNA was extracted using the Qiagen Blood and Tissue Kit. The bacterial 16 S rRNA V4 region was amplified with barcoded 515 F/806R primers and sequenced on an Illumina MiSeq (2 × 300 bp). A mock community ( Microbial Community Standard, ZymoBIOMICS) and negative extraction/PCR controls were included. Amplicon libraries were processed with DADA2 [19] and classified against SILVA v138.2 [20]. Contaminant ASVs were removed using decontam v1.26.0 [21], and sequences assigned to mitochondria, chloroplasts, Archaea, or Eukaryota were excluded. Full bioinformatic parameters are provided in the Supplementary Methods.

### Clinical data analysis

Clinical data were analyzed in IBM SPSS Statistics v27. Parametric or non-parametric tests were applied accordingly; repeated measures were analyzed with linear mixed models (LMM) or generalized estimating equation (GEE) models.

### Microbiota data analysis

Microbiota analyses were performed in R (v4.4.2) using the phyloseq [22] and microeco packages [22, 23]. ASVs were renamed based on the lowest confidently assigned taxon ranked by abundance. Respirotypes were defined by hierarchical clustering (Ward.D2) of Morisita–Horn distances, with the optimal number of clusters determined by the silhouette index.

Alpha diversity was assessed per sample using the Shannon index and relative dominance. Paired T1–T2 differences within each airway niche were tested with Wilcoxon signed-rank tests; effect sizes (Cohen’s d, rank-biserial correlation) and FDR-adjusted q-values (Benjamini–Hochberg) are reported.

For beta diversity, pairwise Morisita–Horn distances were calculated between every niche pair within each patient at T1 and T2, and the paired change in distance was tested per niche pair using Wilcoxon signed-rank tests with FDR correction. Decreasing distances were interpreted as communities becoming more similar (microbial exchange), stable or increasing distances as preserved compartmentalization. The tube community (T3) served as a fixed per-patient reference for tube–niche distances at both T1 and T2. Analyses were restricted to patients with complete sample sets; sensitivity analyses using all available niche pairs are reported in the Supplementary Methods.

### Study endpoints

The primary endpoint was the change in beta diversity (Morisita–Horn distances) between tube-associated, upper-airway, and lower-airway communities from T1 to T2, assessed separately within each intubation group. The contrast between groups was drawn from the pattern of within-group changes that reached statistical significance after FDR correction. Secondary endpoints were changes in within-niche alpha diversity (Shannon index, relative dominance), clinical severity scores (APACHE II, SAPS II, SOFA), and patient survival.

## Results

### Demographic data analysis

Demographic analyses showed no significant differences between the groups in demographic characteristics or comorbidity distribution (Table 1).

**Table 1:** Baseline demographic and clinical characteristics of the study cohort (*n* = 47)

Characteristic	Total (*n* = 47)	ST (*n* = 22)	VT (*n* = 25)	*p*
Sociodemographic				
Male, *n* (%)	36 (76.6)	17 (77.3)	19 (76.0)	1.000
Age, years, mean ± SD	72.0 ± 9.7	69.4 ± 8.5	74.4 ± 10.2	0.330
Weight, kg, mean ± SD	76.2 ± 24.9	77.6 ± 27.8	75.0 ± 22.5	0.831
Height, cm, mean ± SD	172.3 ± 8.9	172.6 ± 8.1	172.0 ± 9.7	0.424
BMI, kg/m², mean ± SD	25.7 ± 8.8	26.3 ± 11.3	25.1 ± 6.2	0.639
Admission diagnoses, *n* (%)				
Pneumonia	33 (70.2)	17 (77.3)	16 (64.0)	0.321
Sepsis	29 (61.7)	12 (54.5)	17 (68.0)	0.241
ARDS severity				0.690
None	22 (46.8)	10 (45.5)	12 (48.0)	
Mild	4 (8.5)	2 (9.1)	2 (8.0)	
Moderate	14 (29.8)	8 (36.4)	6 (24.0)	
Severe	7 (14.9)	2 (9.1)	5 (20.0)	
Pulmonary comorbidities, *n* (%)				
COPD	8 (17.0)	4 (18.2)	4 (16.0)	0.887
Pulmonary embolism	5 (10.6)	1 (4.5)	4 (16.0)	0.170
Chronic bronchitis	3 (6.4)	2 (9.1)	1 (4.0)	0.522
Asthma	2 (4.3)	1 (4.5)	1 (4.0)	1.000
Lung cancer	2 (4.3)	1 (4.5)	1 (4.0)	0.950
Cardiovascular comorbidities, *n* (%)				
Arterial hypertension	33 (70.2)	14 (63.6)	19 (76.0)	0.477
Atrial fibrillation	28 (59.6)	13 (59.1)	15 (60.0)	0.813
Heart failure	24 (51.1)	9 (40.9)	13 (52.0)	0.327
Coronary artery disease	22 (46.8)	9 (40.9)	13 (52.0)	0.578
Myocardial infarction	10 (21.3)	3 (13.6)	7 (28.0)	0.201
Heart valve defect	9 (19.1)	6 (27.3)	3 (12.0)	0.262
Other comorbidities, *n* (%)				
Anemia	32 (68.1)	18 (81.8)	14 (56.0)	0.121
Renal insufficiency	28 (59.6)	14 (63.6)	14 (56.0)	0.713
Other malignancy	26 (55.3)	10 (45.5)	16 (64.0)	0.147
Diabetes mellitus	13 (27.7)	6 (27.3)	7 (28.0)	0.864
Dialysis	10 (21.3)	4 (18.2)	6 (24.0)	0.651

Data presented as *n* (%) or mean ± SD. Continuous variables were compared using the t-test or Mann–Whitney U test as appropriate. Categorical variables were compared using the chi-square test, or Fisher’s exact test where expected cell counts were < 5 (sex, ARDS severity, asthma)

*ST* Standard endotracheal tube, *VT* Venner PneuX^®^ Tube, *BMI* Body mass index, *ARDS* Acute respiratory distress syndrome, *COPD* Chronic obstructive pulmonary disease

### Ventilation parameters

Of 47 patients with ventilation data, 35.3% were mechanically ventilated for more than five days, distributed equally between groups (ST: 9; VT: 9). Mean ventilation duration did not differ significantly (ST: 4.23 ± 1.65 days; VT: 3.48 ± 2.28 days; *p* = 0.152). Linear mixed model analysis across days 1–4 (Table 2) showed significant time effects for FiO₂, PEEP, peak and mean airway pressure, resistance, and compliance, consistent with expected respiratory improvement. No significant group effects were observed. A group × time interaction was detected for tidal volume (*p* = 0.019); this isolated finding across multiple tested parameters should be interpreted cautiously given the pilot sample size.

**Table 2:** Ventilation parameters baseline and across days 1–4 of mechanical ventilation

Parameter	Group	Baseline	Day 1	Day 2	Day 3	Day 4	*p* (time)	*p* (group × time)
p Insp. (mbar) *n* = 130	ST (*n* = 62)	26.4 ± 1.0	24.2 ± 1.0	22.4 ± 1.3	22.6 ± 1.4	25.5 ± 2.0	0.016	0.739
	VT (*n* = 68)	25.0 ± 0.9	24.0 ± 1.0	23.5 ± 1.2	22.0 ± 1.4	24.3 ± 1.7		
Respiratory rate (breaths/min) *n* = 180	ST (*n* = 88)	17.9 ± 1.0	18.7 ± 1.0	18.2 ± 1.0	17.1 ± 1.2	18.9 ± 1.3	0.373	0.777
	VT (*n* = 92)	17.0 ± 0.9	16.6 ± 0.9	17.2 ± 1.0	17.5 ± 1.2	18.1 ± 1.3		
ASB (mbar) *n* = 179	ST (*n* = 87)	9.5 ± 0.8	10.0 ± 0.8	8.9 ± 0.9	9.4 ± 1.0	7.6 ± 1.1	0.110	0.771
	VT (*n* = 92)	8.9 ± 0.8	9.9 ± 0.8	9.9 ± 0.9	9.3 ± 1.0	8.0 ± 1.1		
PEEP (mbar) *n* = 174	ST (*n* = 85)	10.2 ± 0.7	10.2 ± 0.7	8.6 ± 0.7	7.7 ± 0.8	8.8 ± 0.9	0.008	0.717
	VT (*n* = 89)	9.8 ± 0.6	9.1 ± 0.6	8.4 ± 0.7	7.7 ± 0.8	8.0 ± 0.9		
FiO_2_ (%) *n* = 180	ST (*n* = 88)	67 ± 3.5	41 ± 3.6	34 ± 3.7	34 ± 4.4	33 ± 5.0	0.001	0.797
	VT (*n* = 92)	72 ± 3.3	41 ± 3.4	42 ± 3.8	38 ± 4.4	36 ± 5.0		
pPeak (mbar) *n* = 180	ST (*n* = 88)	26.3 ± 1.3	23.7 ± 1.3	20.0 ± 1.3	20.7 ± 1.5	17.8 ± 1.7	0.001	0.446
	VT (*n* = 92)	23.7 ± 1.2	22.8 ± 1.2	20.4 ± 1.3	19.2 ± 1.5	19.1 ± 1.7		
Pmean (mbar) *n* = 180	ST (*n* = 88)	15.6 ± 0.8	15.0 ± 0.8	12.1 ± 0.9	11.9 ± 1.0	10.4 ± 1.1	0.001	0.451
	VT (*n* = 92)	14.5 ± 0.8	13.7 ± 0.8	12.2 ± 0.8	11.7 ± 1.0	11.8 ± 1.1		
Tidal volume (mL) *n* = 179	ST (*n* = 88)	436 ± 32	457 ± 32	533 ± 33	550 ± 39	483 ± 44	0.089	0.019
	VT (*n* = 91)	473 ± 30	569 ± 31	508 ± 34	503 ± 39	571 ± 46		
Resistance (cmH_2_O/(L/s)) *n* = 179	ST (*n* = 87)	19.7 ± 2.1	13.0 ± 2.1	10.0 ± 2.2	11.8 ± 2.6	9.0 ± 3.1	0.001	0.984
	VT (*n* = 92)	19.0 ± 2.0	13.3 ± 2.0	10.0 ± 2.2	10.5 ± 2.6	10.0 ± 2.9		
Compliance (mL/cmH_2_O) *n* = 179	ST (*n* = 87)	34.6 ± 7.9	51.1 ± 8.0	81.5 ± 8.2	65.1 ± 9.8	78.9 ± 11.5	0.001	0.545
	VT (*n* = 92)	35.7 ± 7.4	51.8 ± 7.7	64.5 ± 8.4	61.0 ± 9.8	57.1 ± 11.0		
etCO_2_ (mmHg) *n* = 180	ST (*n* = 88)	41.8 ± 2.5	37.3 ± 2.5	38.2 ± 2.6	34.2 ± 3.0	35.3 ± 3.4	0.117	0.719
	VT (*n* = 92)	39.6 ± 2.3	38.7 ± 2.4	35.7 ± 2.6	34.1 ± 3.0	32.7 ± 3.4		
Horowitz Index (PaO_2_/FiO_2_) *n* = 174	ST (*n* = 86)	32 ± 20	279 ± 21	252 ± 22	293 ± 25	351 ± 29	0.001	0.124
	VT (*n* = 88)	42 ± 19	216 ± 20	246 ± 21	271 ± 25	316 ± 30		

Data presented as mean ± SE from linear mixed model (LMM) estimates. p (time) tests the overall main effect of time across days 1–4; p (group × time) tests whether the trajectory over time differs between groups. n values indicate the number of observations contributing to each parameter, reflecting variable ventilation durations. Baseline values represent measurements at the time of intubation; low baseline Horowitz Index values reflect the emergency nature of intubation in this cohort

*ST* Standard endotracheal tube, *VT* Venner PneuX^®^ Tube, *pInsp*. Inspiratory pressure, *ASB* Assisted spontaneous breathing pressure, *PEEP* Positive end-expiratory pressure, *FiO*_2_ Fraction of inspired oxygen, *pPeak* peak inspiratory pressure, *Pmean* Mean airway pressure, *etCO*_2_ End-tidal carbon dioxide, *Horowitz Index* PaO_2_/FiO_2_ ratio

### Clinical outcome

**Fig. 2:**
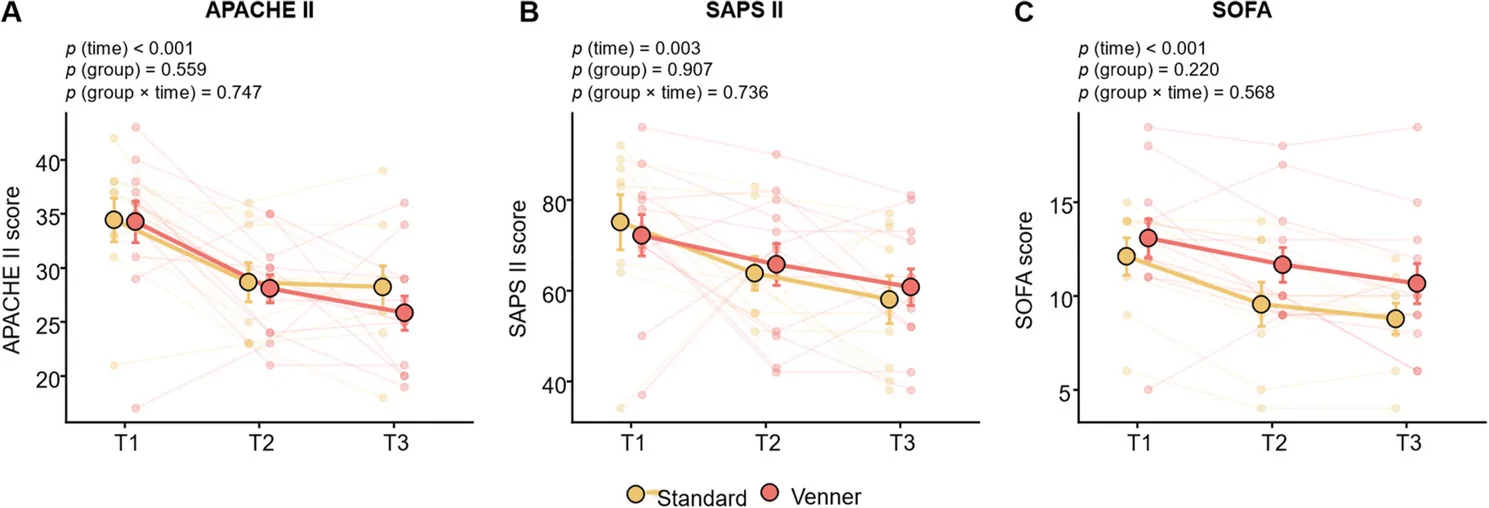
Clinical severity scores over time in the ST and VT groups. Individual patient trajectories and group-level means (± SE) for **A:** the Acute Physiology and Chronic Health Evaluation II (APACHE II) score, **B:** the Simplified Acute Physiology Score II (SAPS II), and **C:** the Sequential Organ Failure Assessment (SOFA) score at the three timepoints: intubation (T1), 72–96 h after intubation (T2), and extubation (T3). Thin semi-transparent lines show individual patient trajectories (orange: standard endotracheal tube, ST, *n* = 9; red: Venner PneuX^®^ Tube, VT, *n* = 12). Bold lines with filled circles show group means; error bars represent standard error. Timepoint positions are slightly offset between groups to improve visibility. *p*-values in each panel are derived from linear mixed models with timepoint and group as fixed effects, their interaction, and a random intercept per patient: *p* (time) tests the overall change across timepoints, *p* (group) tests the average difference between ST and VT across timepoints, *p* (group × time) tests whether the trajectory over time differs between groups

Clinical severity scores (APACHE II, SAPS II, and SOFA) decreased significantly over time in both groups (Fig. 2). Post-hoc comparisons showed significant reductions from T1 to T3 for all three scores (APACHE II Δ = 7.3, *p* = 0.002; SAPS II Δ = 14.3, *p* = 0.007; SOFA Δ = 2.9, *p* = 0.001), with earlier reductions by T2 for APACHE II and SOFA. No significant group effects or time × group interactions were observed. Survival rates were comparable between groups (ST: 77.8%; VT: 83.3%; Fisher’s exact *p* = 0.586), although the sample size was too small for meaningful inference.

### Comparable anti-infective therapy, timing of first pathogen detection, and site of infection between groups

Overall, anti-infective therapy, the timing of first pathogen detection, and the site of infection were comparable between groups. From day 1 onward, all patients received anti-infective treatment. No significant differences were observed in the timing of first pathogen detection: in the full cohort, no pathogen was detected in 31.8% of ST patients and 16.0% of VT patients, while first detection occurred most frequently on day 3 (18.2% ST vs. 24.0% VT) (Table S1). Similarly, the pulmonary site was the most common focus of infection in both groups, affecting approximately half of all patients (45.5% ST vs. 48.0% VT), with no significant differences across infection site categories (Table S2).

### Alpha diversity dynamics in different airway niches after intubation

**Fig. 3:**
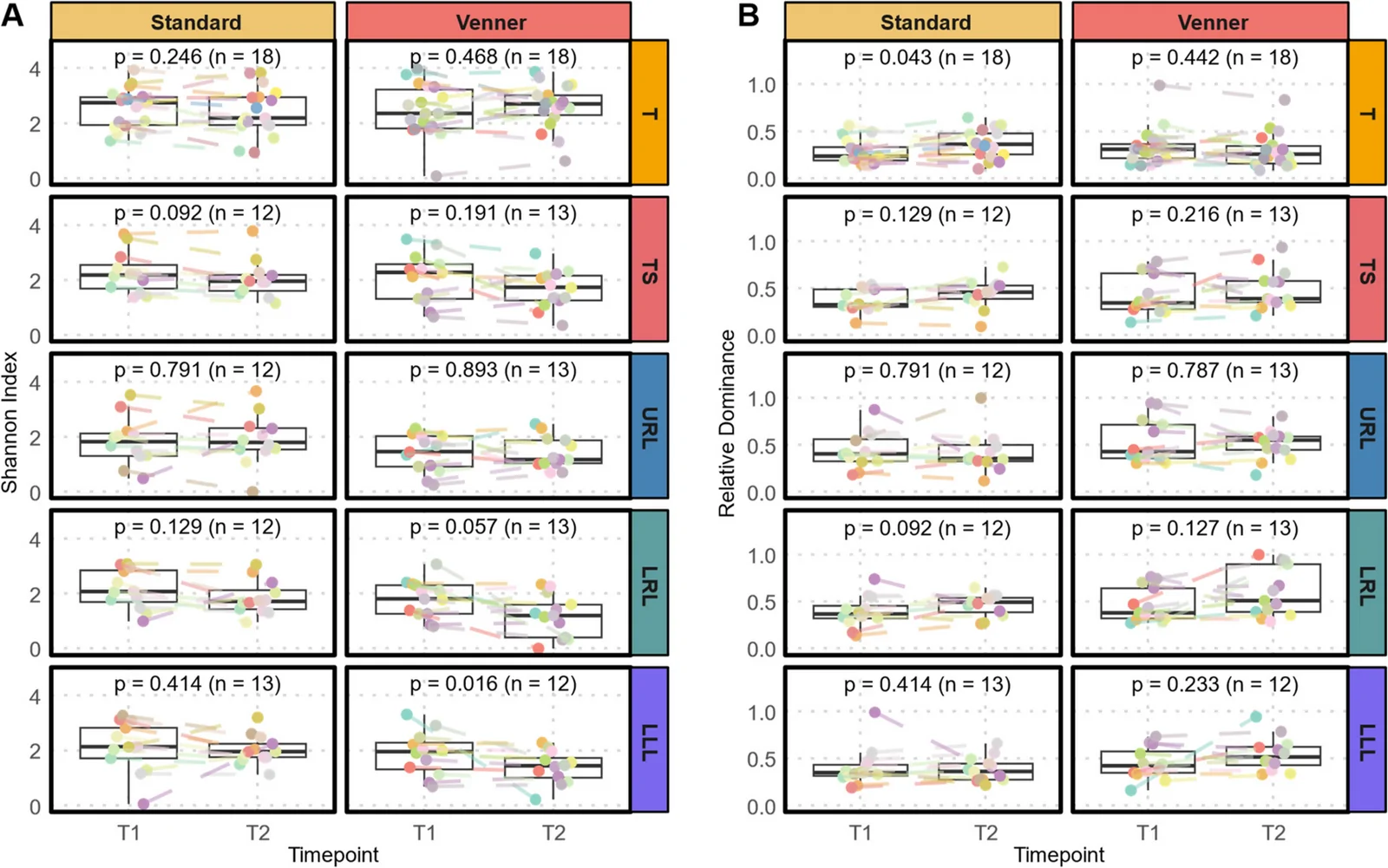
Comparison of microbial alpha diversity measures across different niches and timepoints. **A**: Shannon diversity index, **B**: Relative dominance. Samples were collected from five niches (T: Throat, TS: Trachea, URL: Upper Right Lung, LRL: Lower Right Lung, LLL: Lower Left Lung), before intubation (T1) and after intubation (T2). Each patient is represented by connected points to show paired changes between timepoints. Paired differences were tested using Wilcoxon signed-rank tests, with raw p-values displayed above each comparison. The n indicates the number of paired samples used for each comparison

Alpha diversity was compared between T1 and T2 across all airway niches and both intubation methods (Fig. 3). Changes in the upper airway niches were minor. In the throat, Shannon diversity remained stable, with only the ST group showing an increase in dominance. In the trachea, Shannon diversity declined slightly after ST intubation and more clearly after VT intubation, alongside modest increases in dominance in both groups. In the upper lung region (URL), neither metric changed meaningfully. In contrast, the lower lobes exhibited the strongest alterations. In the lower right lobe (LRL), Shannon diversity decreased in both groups with parallel increases in dominance. The most marked change occurred in the lower left lobe (LLL), where diversity remained stable after ST intubation but declined substantially after VT intubation, although this was not significant after correction.

### Beta diversity changes across airway niches following intubation

**Fig. 4:**
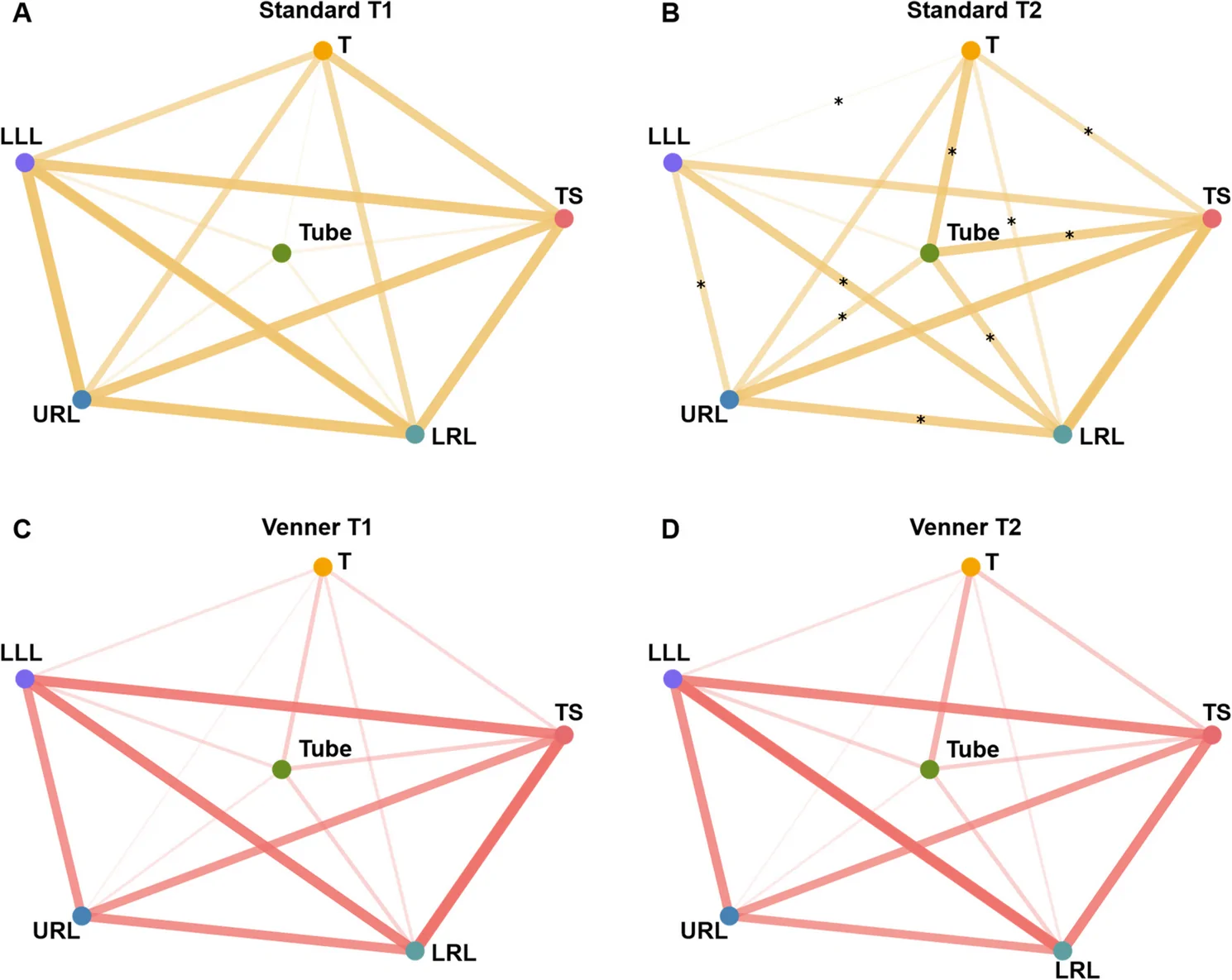
Beta Diversity Similarity Graph representation of Morisita-Horn distances between airway niches before and after intubation (**A: **ST before intubation, **B : **ST after intubation, **C : **VT before intubation, **D: **VT after intubation). Nodes represent airway niches (T: Throat, TS: Trachea, URL: Upper Right Lung, LRL: Lower Right Lung, LLL: Lower Left Lung), and edges indicate the mean pairwise microbial community distance between niches. Edge thickness and transparency are inversely proportional to distance, with thicker and more opaque edges representing more similar communities. Stars (*) at Timepoint 2 indicate niche-niche pairs whose community composition changed significantly after intubation (adjusted *p* < 0.05). This analysis was based only on patients with complete sample sets (orange: standard endotracheal tube, ST, *n* = 9; red: Venner PneuX^®^ Tube, VT, *n* = 12)

Beta diversity was compared between T1 and T2 across airway niches and intubation methods (Fig. 4). In the ST group, airway communities became more similar to the final tube-associated community after intubation, reflected by reduced niche–tube distances [TS–Tube: 0.55 ± 0.24 → 0.30 ± 0.27 (*p* = 0.001); T–Tube: 0.61 ± 0.22 → 0.30 ± 0.21 (*p* = 0.004); LRL–Tube: 0.54 ± 0.26 → 0.36 ± 0.28 (*p* = 0.019)]. At the same time, lung–lung distances increased, particularly for LLL–LRL (0.08 ± 0.17 → 0.31 ± 0.38; *p* = 0.003), LLL–URL (0.09 ± 0.19 → 0.40 ± 0.09; *p* < 0.001), and LRL–URL (0.10 ± 0.24 → 0.30 ± 0.32; *p* = 0.011). Lung–throat dissimilarity also increased, such as LLL–T (0.30 ± 0.23 → 0.61 ± 0.29; *p* < 0.001) and LRL–T (0.29 ± 0.22 → 0.50 ± 0.30; *p* = 0.026). In the VT group, tube–lung and lung–lung distances remained largely unchanged; only LRL–TS (0.07 ± 0.08 → 0.28 ± 0.40; *p* = 0.005) showed a difference that did not remain significant after correction (q = 0.080). At baseline (T1), microbial community distances did not differ between groups (Figure S3). Delta distances (T2 -T1) were larger in the ST group than in the VT group, particularly for tube–airway pairs, but no niche pair reached significance after correction (Table S3, Figure S4).

### Microbial community clustering across patients and niches

**Fig. 5:**
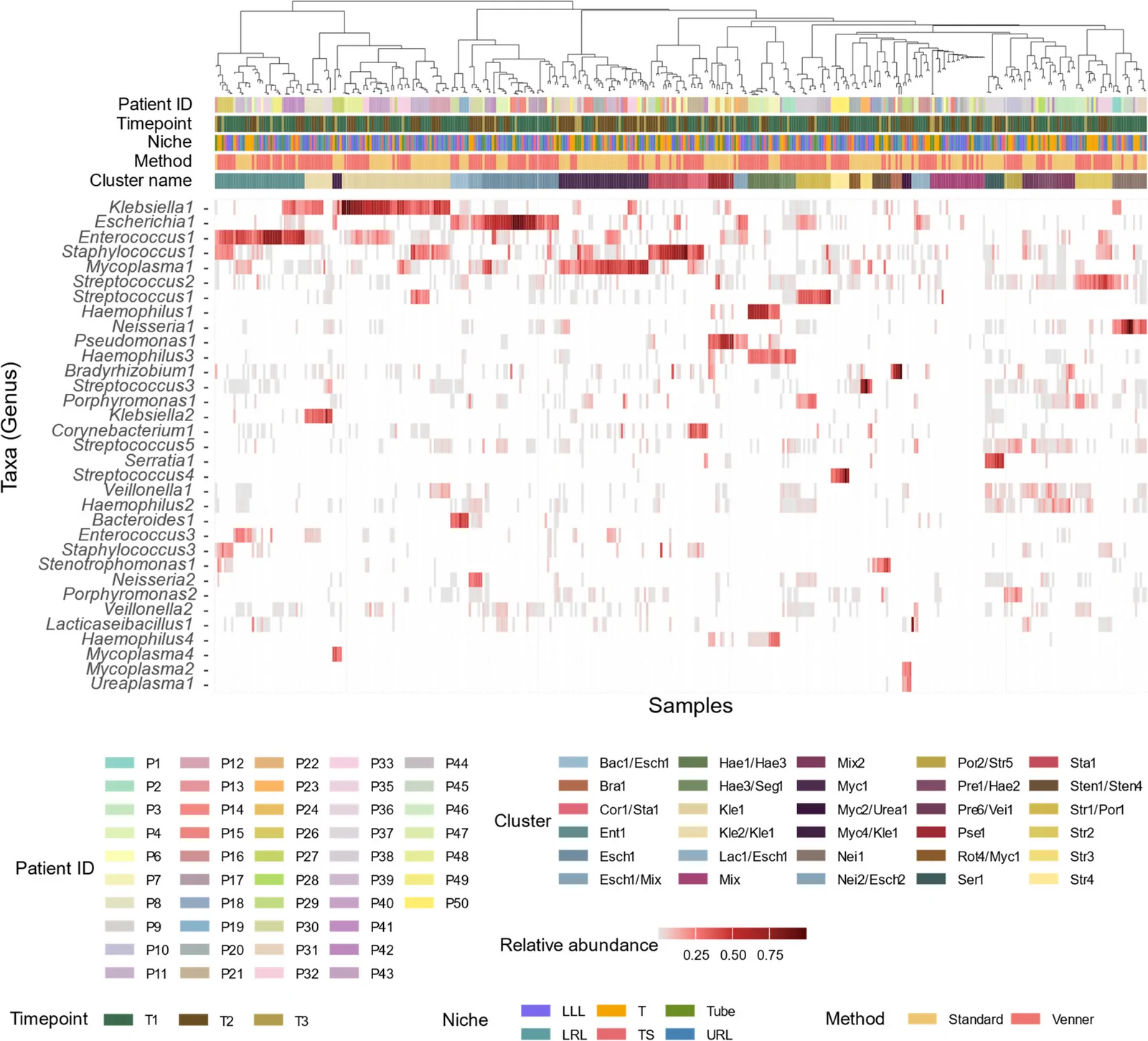
Hierarchical clustering of airway microbiota samples collected from patients across multiple niches (LLL = Lower Left Lobe, LRL = Lower Right Lobe, URL = Upper Right Lobe, TS = Trachea, T = Throat). Two sampling timepoints were included per patient per niche: T1 (baseline) and T2 (post-intubation). Tube samples, taken after intubation, are labelled as T3 for visualization. The dendrogram represents sample similarity based on the Morisita-Horn dissimilarity matrix. Adjacent annotation bars indicate patient identity, sample timepoint (T1 = Before intubation, T2 = After intubation, T3 = Tube sample), niche, intubation method, and cluster assignment. Cluster (Respirotype) names (Kle1 = Klebsiella, Myc1 = Mycoplasma, Nei1 = Neisseria, Bac1/Esch1 = Bacteroides/Escherichia, Rot4/Myc1 = Rothia/Mycoplasma, Str3 = Streptococcus, Myc2/Urea1 = Mycoplasma/Ureaplasma and Nei2/Esch2 = Neisseria/Escherichia) correspond to the most abundant bacterial taxa within each group. The heatmap displays the relative abundance of the top taxa across samples, with colour intensity reflecting increasing abundance. All available samples (*n* = 404) were used for this visualisation

Hierarchical clustering of Morisita–Horn distances across all samples identified 30 microbial clusters (Fig. 5). The prominent cluster Klebsiella1 occurred in 8 patients across niches, with higher prevalence at T1 (59.6% vs. 36.2%) and in ST vs. VT samples (15.7% vs. 8.4%; Figure S5, Figure S6). Mycoplasma1 was detected in 14 patients, mainly at T2 (66.7%), and more often in ST than VT samples (15.7% vs. 4.2%). Most clusters were independent of timepoint or intubation method and occurred in few patients, indicating no consistent patterns.

## Discussion

This study assessed whether the VT system reduces microbial exchange between upper and lower airway compartments compared with a standard endotracheal tube. Overall microbiota composition remained similar between groups, but community dynamics differed. In the ST group, Morisita–Horn distances between the tube and both the tracheal and lower-airway compartments decreased significantly from T1 to T2, meaning that lower-airway communities became more similar to the final tube-associated community. At the same time, distances between lung regions and between lung and throat increased, meaning these compartments diverged from each other, reflecting loss of the coherent airway ecosystem present at baseline. This finding is consistent with tube-driven disruption of airway compartmentalization, as also suggested in previous studies [9, 11, 24]. In the VT group, none of the tube–niche or inter-niche distances changed significantly, which may indicate more stable ecological relationships between airway compartments, though this observation alone does not establish preserved compartmentalization. The contrast between groups is consistent with the hypothesis that the VT system’s cuff seal and subglottic suction reduce microbial flux between compartments and, by doing so, may reduce the risk of ventilator-associated pneumonia from microaspiration [12, 16]. A formal between-group comparison showed larger shifts in the ST group, particularly for tube-airway pairs, but did not reach significance, and the findings should therefore be considered exploratory pending confirmation in larger studies.

Both groups showed a decline in Shannon diversity over time in the lower airways, with parallel increases in relative dominance. The decline was most pronounced in the VT group. Mechanical ventilation inherently disturbs airway ecology, and loss of diversity under ventilation [10] has been associated with worse outcomes in several cohorts [9, 24]. Any endotracheal tube contributes to this disturbance by bypassing natural airway defenses and altering the separation between upper and lower airway compartments [11], and both groups in our cohort were additionally exposed to ICU-wide factors that reduce airway diversity, including antibiotics [9, 25], mechanical ventilation [26], and altered mucus clearance [27]. A decline in distal alpha diversity is therefore expected regardless of tube type. The more pronounced decline in alpha diversity, observed in the VT group, is consistent with a more effective barrier function [12]. Mechanical ventilation is associated with reduced microbial diversity [10]. In this context, reduced microbial exchange across the cuff with integrated subglottic suction may limit the inflow of taxa to the distal lung, thereby favoring competitive outgrowth of better-adapted resident organisms and resulting in lower richness and higher dominance [5]. Whether this additional decline in diversity represents beneficial reduction of pathogen translocation, undesirable loss of protective commensals, or both, cannot be resolved from the present data and warrants further investigation.

Cluster analysis showed no group-level significant differences, but some patient-level patterns are worth noting. The Mycoplasma1 cluster, which colonizes at T1 primarily the upper airways, appeared more often in ST samples at T2. This correlates with the beta diversity pattern of translocation into the lower airways [10, 14]. The Klebsiella1 cluster was more prevalent at baseline than at T2, and more common in the ST group overall, likely reflecting baseline colonization differences rather than tube-mediated effects [14, 28].

Both groups were clinically comparable. Demographics, comorbidities, and baseline severity were well balanced. Ventilation duration did not differ significantly and an equal number of patients in each group were ventilated beyond five days. Likewise, no relevant between-group differences were observed with respect to the site of infection or anti-infective therapy. Approximately half of the patients had a pulmonary focus of infection, and the distribution of administered anti-infective agents was similar across groups. All patients received anti-infective therapy from the time of intervention onward; because microbiological pathogen detection was often unsuccessful or only established between study days 2 and 4, initial treatment was predominantly empirical. Ventilation parameters and severity scores (APACHE II, SAPS II, SOFA) improved over time without group-specific effects, suggesting that respiratory trajectories reflected the underlying illness rather than the tube. Survival did not differ between groups, though the sample size was too small for any meaningful inference about mortality.

VAP was not an endpoint of this study and no dedicated VAP diagnostics were performed, so the reductions in VAP incidence reported in earlier studies of the VT system [16, 17] could not be evaluated directly. However, the microbiota findings provide a complementary line of evidence. Previous assessments of the VT system’s protective effect have relied on benchtop cuff-leakage tests [12], which measure whether a standardized test fluid passes the inflated cuff under controlled conditions. These tests cannot capture the complexity of real airway secretions or the cumulative microaspiration during ventilation. Microbiota sampling across paired airway compartments, by contrast, records the in vivo result of this process (the actual microbial exchange between compartments over time) [9, 24] and may serve as an endpoint that sits between in vitro leakage testing and clinical VAP surveillance for evaluating airway device performance.

The study’s randomized design, paired multi-niche sampling across airway compartments over time, and standardized protocols represent methodological strengths. Nonetheless, limitations remain. This was a pilot study; the single-center design and small cohort size (9 ST and 12 VT patients with complete microbiota data) limit the generalizability of the findings. Although anti-infective therapy, concomitant infections, and the site of infection were recorded as potential determinants of microbiome composition [15], the sample size was too small to assess their impact reliably or to adjust for potential confounders. The inclusion criterion of acute respiratory failure is heterogeneous [29], and patients in both groups had varied underlying etiologies. The study assessed only the bacterial component of the airway microbiota through 16 S rRNA sequencing. Because the endotracheal tube was sampled only at extubation, it represents a cumulative endpoint community shaped over the entire ventilation period rather than a time-matched reference. Consequently, the direction of microbial exchange between the tube and airway compartments cannot be determined from these data, as decreasing tube–airway distances may reflect colonization of airway compartments from the tube, colonization of the tube from airway compartments, or both simultaneously. Implementation of the VT system required a training phase during which occasional re-intubations occurred. Finally, the study design cannot distinguish whether the observed differences reflect the VT system as a whole or specific components such as the cuff-pressure monitoring, subglottic suction or biofilm-resistant coating.

## Conclusion

In this pilot study, the VT system was associated with preserved compartmentalization between tube-associated, upper-airway, and lower-airway communities during mechanical ventilation, whereas standard intubation showed progressive microbial convergence between these compartments. These findings are consistent with the proposed microaspiration-reducing mechanism of the VT system and provide exploratory biological support for the previously reported reduction in VAP incidence. Beyond the comparison between tube designs, these results suggest that lung microbiota sampling may serve as a physiologically meaningful endpoint for evaluating airway device performance. Larger multicenter trials are needed to confirm these observations and to link microbial-exchange signatures to clinical outcomes such as VAP incidence and ventilator-associated complications.

## Supplementary Information

Supplementary Material 1.

## Data Availability

All analysis code and data used in the study are available at https://github.com/patrickDsal/tubomic-airway-microbiota.
