# Supplementary material for: Airway microbial compartmentalization under mechanical ventilation: a randomized pilot study comparing an endotracheal tube with suction and continuous cuff pressure control (Venner PneuX®) to standard intubation
Source: Respir Res. 2026 Aug 1;27:303. doi: 10.1186/s12931-026-03849-2 (PMC13452131; doi:10.1186/s12931-026-03849-2)
Supplement: Supplementary file 1 [file 12931_2026_3849_MOESM1_ESM.docx]

**Supplementary Material**

**Tables**

**Table S1. Timing of First Pathogen Detection in Standard Endotracheal Tube versus Venner PneuX® Tube Groups**

| Variable | Group | Baseline | No pathogen detected | Day 1 | Day 2 | Day 3 | Day 4 |
| --- | --- | --- | --- | --- | --- | --- | --- |
| First pathogen detection  (all patients)  n=47 | ST (n=22) | 2  9,1% | 7  31,8% | 1  4,5% | 4  18,2% | 3  13,6% | 5  22,7% |
|  | VT (n=25) | 4  16% | 4  16% | 4  16% | 6  24% | 5  20% | 2  8% |
|  | p-value | 1.000 | 0,480 | 0,353 | 0,730 | 0,706 | 0,228 |
| First pathogen detection (complete dataset)  n=21 | ST  (n=9) | 1  11% | 1  11% | 0  0% | 3  33% | 2  22% | 2  22% |
|  | VT (n=12) | 2  17% | 0  0% | 4  33% | 3  25% | 1  8% | 2  17% |
|  | p-value | 1,000 | 0,429 | 0,204 | 1,000 | 0,553 | 1,000 |

Data presented as n (%). ST, standard endotracheal tube; VT, Venner PneuX® Tube. The timing of first pathogen detection was compared between groups. Results were recorded at baseline and on days 1-4 for all patients (n=47) as well as for the complete dataset (n=21). Categorical variables were compared using Fisher's exact test, as expected cell counts were <5.

**Table S2. Site of Infection in Standard Endotracheal Tube versus Venner PneuX® Tube Groups**

|  | Group | Pulmonary | Abdominal | UNK | BS | Other | Multiple sites |
| --- | --- | --- | --- | --- | --- | --- | --- |
| Site of infection (all patients) n=47 | ST  (n=22) | 10  45,5% | 3  13,6% | 7  31,8% | 0  0% | 1  4,5% | 1  4,5% |
|  | VT  (n=25) | 12  48% | 0  0% | 4  16% | 4  16% | 2  8% | 3  12% |
|  | p-value | 0,777 | 0,101 | 0,159 | 0,110 | 0,278 | 0,159 |
| Site of infection (complete dataset)  n=21 | ST  (n=9) | 6  66,7% | 1  11,1% | 1  11,1% | 0  0% | 1  11,1% | 0  0% |
|  | VT  (n=12) | 6  50% | 0  0% | 0  0% | 2  16,7% | 2  16,7% | 2  16,7% |
|  | p-value | 0,661 | 0,429 | 0,429 | 0,486 | 1,000 | 0,486 |

Data presented as n (%). ST, standard endotracheal tube; VT, Venner PneuX® Tube; UNK, unknown; BS, bloodstream. The site of infection was compared between groups for all patients (n=47) and for the complete dataset (n=21). Categorical variables were compared using Fisher's exact test, as expected cell counts were <5.

Table S3. Between-group comparison of T2−T1 changes in niche-pair Morisita–Horn distances

| **Niche Pair** | **n ST** | **n VT** | **Delta ST**  **(mean ± SD)** | **Delta VT**  **(mean ± SD)** | **p** | **q** | **Sig** |
| --- | --- | --- | --- | --- | --- | --- | --- |
| **LLL_LRL** | 9 | 12 | 0.24 ± 0.36 | 0.07 ± 0.44 | 0.345 | 0.584 | ns |
| **LLL_T** | 9 | 12 | 0.31 ± 0.29 | 0.09 ± 0.41 | 0.159 | 0.554 | ns |
| **LLL_TS** | 9 | 12 | 0.21 ± 0.41 | 0.15 ± 0.33 | 0.690 | 0.941 | ns |
| **LLL_Tube** | 9 | 12 | 0.04 ± 0.41 | 0.07 ± 0.43 | 0.854 | 0.947 | ns |
| **LLL_URL** | 9 | 12 | 0.31 ± 0.39 | 0.11 ± 0.49 | 0.314 | 0.584 | ns |
| **LRL_T** | 9 | 12 | 0.21 ± 0.29 | 0.12 ± 0.42 | 0.615 | 0.922 | ns |
| **LRL_TS** | 9 | 12 | 0.08 ± 0.37 | 0.22 ± 0.33 | 0.350 | 0.584 | ns |
| **LRL_Tube** | 9 | 12 | -0.18 ± 0.39 | 0.09 ± 0.34 | 0.092 | 0.461 | ns |
| **LRL_URL** | 9 | 12 | 0.19 ± 0.34 | 0.17 ± 0.44 | 0.904 | 0.947 | ns |
| **TS_Tube** | 9 | 12 | -0.25 ± 0.32 | 0.11 ± 0.25 | 0.005 | 0.074 | ns |
| **TS_URL** | 9 | 12 | 0.13 ± 0.36 | 0.14 ± 0.44 | 0.947 | 0.947 | ns |
| **T_TS** | 9 | 12 | 0.22 ± 0.28 | 0.04 ± 0.37 | 0.221 | 0.554 | ns |
| **T_Tube** | 9 | 12 | -0.31 ± 0.35 | -0.01 ± 0.33 | 0.042 | 0.315 | ns |
| **T_URL** | 9 | 12 | 0.11 ± 0.32 | 0.09 ± 0.54 | 0.930 | 0.947 | ns |
| **Tube_URL** | 9 | 12 | -0.12 ± 0.38 | 0.11 ± 0.45 | 0.212 | 0.554 | ns |

Negative delta values indicate convergence; positive values indicate divergence. P-values from the Timepoint x Method interaction term (Distance ~ Timepoint x Method + (1|Patient)) with Benjamini–Hochberg FDR correction. T = Throat, TS = Trachea, URL = Upper Right Lobe, LLL = Lower Left Lobe, LRL = Lower Right Lobe.

**Figures**

Figure S1. A: Overview of the number of samples (S), amplicon sequence variants (ASVs; A), and sequencing reads (R) retained after each filtering step. B: Raincloud plot showing the distribution of sequencing reads per sample (log-transformed). The dashed vertical line indicates 2,300 reads. Two biological samples did not pass quality filtering and were removed from the final analysis. C: Bar chart showing the relative abundances of taxa in mock community samples. Expected compositions of the mock community are compared to the observed abundances of positive control samples after sequencing and data processing.

Figure S2. Sample availability across patients, time points and anatomical niches. Each dot represents one available sample; absence of a dot indicates a missing sample. Dot color indicates the intubation method (Standard or Venner). Panels (No/Yes) separate patients according to whether they belonged to the full dataset (9 full sets for Standard; 12 for Venner). Abbreviations: LLL = lower left lobe; LRL = lower right lobe; URL = upper right lobe; TS = trachea; T = throat.

Figure S3. Comparison of Morisita–Horn distances between airway niche pairs under Standard and Venner intubation sampled before intubation (T1). Each panel shows distances for one airway niche pair across patients. Boxplots represent the distribution per method, while coloured points reflect individual patients. Reported p-values are from unpaired Wilcoxon rank-sum tests comparing Standard vs. Venner for each niche pair. Lower Morisita–Horn values indicate greater similarity between microbial communities, whereas higher values indicate greater dissimilarity. Abb.: T = Throat, TS = Trachea, URL = Upper Right Lobe, LLL = Lower Left Lobe, LRL = Lower Right Lobe. All available samples were considered for this analysis.

Figure S4. Between-group comparison of T2-T1 changes in niche-pair Morisita–Horn distances. Each panel shows a specific airway niche pair, with the change in pairwise community dissimilarity (Δ Distance = T2 -T1) compared between the Standard and Venner intubation groups. Boxplots indicate the distribution across patients, while coloured points represent individual patient values. Negative delta values indicate that communities became more similar after intubation, whereas positive values indicate increased dissimilarity. Reported p-values are from a linear mixed-effects model (Distance ~ Timepoint x Method + (1|Patient)), reflecting the Timepoint x Method interaction term with Benjamini–Hochberg FDR correction. Analysis was restricted to patients with full sample sets (Standard: n = 9, Venner: n = 12). Abb.: T = Throat, TS = Trachea, URL = Upper Right Lobe, LLL = Lower Left Lobe, LRL = Lower Right Lobe.

Figure S5. Changes in microbial cluster composition across timepoints for individual patients intubated with the Standard method. Each panel represents a single patient, with clusters at Timepoint 1 (T1) and Timepoint 2 (T2) displayed as parallel sets. Each flow corresponds to niches transitioning between clusters. Colours indicate niche identity (T = Throat, TS = Trachea, URL = Upper Right Lobe, LLL = Lower Left Lobe, LRL = Lower Right Lobe). Only patients with full sample sets were considered.

Figure S6. Changes in microbial cluster composition across timepoints for individual patients intubated with the Venner method. Each panel represents a single patient, with clusters at Timepoint 1 (T1) and Timepoint 2 (T2) displayed as parallel sets. Each flow corresponds to niches transitioning between clusters. Colours indicate niche identity (T = Throat, TS = Trachea, URL = Upper Right Lobe, LLL = Lower Left Lobe, LRL = Lower Right Lobe). Only patients with full sample sets were considered.

Figure S7. Paired niche–niche Morisita–Horn distances in the Standard group before and after intubation. Each panel shows a specific airway niche pair, with distances compared between baseline (T1) and post-intubation (T2). Boxplots indicate the distribution across patients, while coloured points and dashed lines trace individual patient trajectories. Lower Morisita–Horn values indicate greater similarity between microbial communities, whereas higher values indicate greater dissimilarity. Reported p-values are from paired Wilcoxon signed-rank tests (uncorrected). Abb.: T = Throat, TS = Trachea, URL = Upper Right Lobe, LLL = Lower Left Lobe, LRL = Lower Right Lobe. All available samples were considered for this analysis.

Figure S8. Paired niche–niche Morisita–Horn distances in the Venner group before and after intubation. Each panel shows a specific airway niche pair, with distances compared between baseline (T1) and post-intubation (T2). Boxplots indicate the distribution across patients, while coloured points and dashed lines trace individual patient trajectories. Lower Morisita–Horn values indicate greater similarity between microbial communities, whereas higher values indicate greater dissimilarity. Reported p-values are from paired Wilcoxon signed-rank tests (uncorrected). Abb.: T = Throat, TS = Trachea, URL = Upper Right Lobe, LLL = Lower Left Lobe, LRL = Lower Right Lobe. All available samples were considered for this analysis.

**Supplementary Methods**

**Study Design and Participants**

This prospective, randomized, single-center pilot study was registered at the German Clinical Trials Register (DRKS00029176) and conducted in the Surgical Intensive Care Unit of Heidelberg University Hospital between November 2022 and May 2024.

Inclusion criteria:

- Age ≥ 18 years
- Acute respiratory failure requiring invasive mechanical ventilation
- Written informed consent from the patient or a legal representative

Exclusion criteria:

- Pregnancy
- Age <18 years
- Refusal to participate

Randomization was performed using sealed envelopes stored on the airway management trolley, with 1:1 allocation to the ST or VT group (n = 25 per group).

**Patient accounting**

Of the 50 randomized patients, 3 were excluded before analysis (withdrew consent after randomization). This left 47 patients for demographic and clinical analyses (Table 1). Of these, 21 patients (9 ST, 12 VT) had complete microbiota sample sets across all airway niches at both T1 and T2 and were included in the primary microbiota analysis. Reasons for incomplete microbiota datasets in the remaining 26 patients included extubation before T2, insufficient sample volume or failed DNA extraction. A detailed flow diagram is provided in Figure 1B.

**Clinical Data Collection**

Demographic, clinical, and laboratory data were retrieved from electronic medical records using the COPRA (Copra System GmbH) and i.s.h med (Integrated Solution for Healthcare Medicine) software systems. Ventilation parameters were recorded immediately after intubation and then every 24 hours for four days. Routine laboratory values, microbiological diagnostics, antibiograms, and anti-infective therapies were documented throughout the observation period. Clinical severity scores (APACHE II, SAPS II, SOFA) were calculated at intubation (T1), after four days of ventilation (T2), and at extubation (T3).

**Sample Collection**

Because microbial communities across airway compartments carry signatures of the microaspiration process, we sampled at five anatomical sites to capture potential compartmental exchange. Sampling followed a standardized protocol to minimize cross-contamination between anatomical sites. At T1 (intubation) and T2 (after four days of mechanical ventilation), samples were obtained in the following sequence:

1. Throat swab (T)
2. Aspiration of tracheal secretions (TS)
3. Bronchoalveolar wash (BW), performed sequentially in the right upper lobe (URL), right lower lobe (LRL), and left lower lobe (LLL) using 10 mL of 0.9% NaCl per site

At T3 (extubation), a swab from the distal tip of the endotracheal tube was collected to characterize the tube-associated microbial community. Because the tube was only sampled at extubation, a single tube sample per patient served as the reference for tube–niche comparisons at both T1 and T2.

One aliquot per site was processed by the clinical microbiology laboratory; the remaining material was stored for research.

**Bioinformatic Processing**

Amplicon libraries were processed with DADA2 (v1.34.0) in R (v4.3.3). Processing steps and parameters:

- **Primer trimming:** Primers were removed using Cutadapt (version 4.4) with linked adapters, discarding read pairs where primers were not detected (--discard-untrimmed), minimum length 200 bp, and minimum overlap 10 bp.
- **Quality filtering and trimming:** Forward reads truncated to **220 bp;** reverse reads truncated to **200 bp**; maximum expected errors maxEE = (2, 2); truncQ = 2; PhiX reads removed (rm.phix = TRUE).
- **Error rate learning:** learnErrors() with default parameters, using multithreading (multithread = TRUE).
- **Denoising:** dada() with pooling option **"independent"** (pool = FALSE), processing each sample independently.
- **Paired-end merging:** mergePairs() with default minimum overlap of 12 bp.
- **Chimera removal:** removeBimeraDenovo() method = "consensus", applied per sequencing run prior to merging sequence tables across runs.
- **Taxonomic assignment** against the SILVA reference database (version 138.2) using naïve Bayesian classification (assignTaxonomy()) with a minimum bootstrap confidence of 50 (default).

***Decontamination and filtering:***

- Contaminant ASVs were identified and removed using the decontam R package (v1.26.0) with a combined frequency- and prevalence-based approach (isContaminant(method = "combined")), using a threshold of **0.1** (default).
- Sequences assigned to mitochondria, chloroplasts, Archaea, or Eukaryota were excluded from downstream analyses.

***Sequencing depth and normalization:***

- Samples with fewer than **2300** reads were excluded from downstream analyses (Figure S1).
- For diversity analyses, samples were **analyzed without rarefaction**.

**Clinical Data Analysis**

Descriptive analyses were performed using IBM SPSS Statistics (version 27; IBM Corp., Armonk, NY, USA). Normality of continuous variables was assessed using the Shapiro–Wilk and Kolmogorov–Smirnov tests, complemented by visual inspection of Q–Q and P–P plots.

- Normally distributed data: parametric tests (t-test, ANOVA)
- Non-normally distributed data: Mann–Whitney U test, Wilcoxon signed-rank test
- Categorical data: Chi-square test or Fisher's exact test as appropriate
- Repeated measures: analyzed using linear mixed models (LMM) with time as a within-subject factor and intubation group as a between-subject factor, and, alternatively, generalized estimating equations (GEE, logit link) with clustering by patient (fixed factors: group, day, group x day).

**Recording of Ventilatory Parameters**

Mechanical ventilation was initiated in BIPAP mode in all patients. Subsequent weaning followed the standardized intensive care unit protocol and was based on the patient's individual clinical condition, including spontaneous awakening trials (SATs), spontaneous breathing trials (SBTs), and a stepwise transition to CPAP until extubation. All ventilatory parameters, including airway resistance and respiratory compliance, were recorded by the Dräger Evita ventilator and routinely documented. The compliance values reported in this study therefore represent dynamic respiratory compliance.

**Microbiota Data Analysis**

All microbiota analyses were performed in R (version 4.4.2) using the phyloseq (version 1.54) and microeco (version 1.16) packages. ASVs were renamed according to the lowest confidently assigned taxon, ranked by abundance within each taxonomic level.

Respirotype clustering:

Samples were clustered hierarchically using Ward.D2 linkage on a Morisita–Horn distance matrix. The optimal number of clusters was selected using the silhouette index. Resulting clusters were labeled according to their dominant taxon (or two most abundant taxa, where no single taxon clearly dominated).

Alpha diversity:

Shannon diversity (capturing both richness and evenness) and relative dominance (proportion of the most abundant taxon) were computed per sample. For each patient and each airway niche, paired T1 and T2 values were compared using Wilcoxon signed-rank tests to evaluate the effect of intubation method (ST vs. VT). Effect sizes were calculated as Cohen's d and rank-biserial correlation. Both raw p-values and FDR-adjusted q-values (Benjamini–Hochberg) are reported. Analyses included all matched T1–T2 patient–niche pairs (n = 272).

Beta diversity:

Pairwise sample dissimilarity was quantified using Morisita–Horn distances. For each patient, pairwise Morisita–Horn distances were computed between every combination of airway niches (T, TS, URL, LRL, LLL) and between each niche and the tube-associated community (sampled at T3). These niche-pair distances were calculated separately at T1 and T2. For each niche pair, the paired change in distance (T2 – T1) across patients was tested using Wilcoxon signed-rank tests with Benjamini–Hochberg FDR correction. Decreasing distances from T1 to T2 were interpreted as airway communities becoming more similar (consistent with microbial exchange across compartments), while stable or increasing distances were interpreted as preserved compartmentalization. Because the tube was sampled only at T3, the tube community represents a cumulative endpoint community shaped over the entire ventilation period rather than a time-matched reference; accordingly, tube–niche distances reflect similarity to this final tube-associated state rather than contemporaneous convergence at each timepoint. Contemporaneous tube sampling during active ventilation was not performed, as this would have required interruption of ventilation.

*Between-group comparison of temporal dynamics:*

To formally test whether the magnitude of change in niche-pair distances differed between intubation groups, two complementary approaches were applied. First, for each patient and each niche pair, a delta value was computed as the difference between T2 and T1 distances (ΔDistance = T2 − T1). Delta values were compared between ST and VT groups using unpaired Wilcoxon rank-sum tests with Benjamini–Hochberg FDR correction. Second, a linear mixed-effects model was fitted per niche pair with distance as the outcome, timepoint (T1/T2) and intubation method (ST/VT) as fixed effects, and patient as a random intercept: Distance ~ Timepoint x Method + (1|Patient). The p-value for the Timepoint x Method interaction term was used as the formal between-group test of temporal change. Both analyses were performed on the full set dataset and on all available samples. Models were fitted using the lme4 and lmerTest packages; p-values were obtained using Satterthwaite approximation.

Sensitivity analysis:

Primary beta diversity analyses were restricted to patients with complete sample sets (n = 9 ST, n = 12 VT; Figure S2). To validate these findings and increase statistical power, analyses were repeated using all available niche pairs regardless of whether a patient contributed a complete set across all five niches and both timepoints. This increased the effective sample size from 9 to 14 for certain ST niche-pair combinations and from 12 to 17 for certain VT combinations. Results were consistent across both approaches; sensitivity analysis results are reported in Figures S7, S8.
